# Early Myeloid Dendritic Cell Dysregulation is Predictive of Disease Progression in Simian Immunodeficiency Virus Infection

**DOI:** 10.1371/journal.ppat.1001235

**Published:** 2010-12-23

**Authors:** Viskam Wijewardana, Adam C. Soloff, Xiangdong Liu, Kevin N. Brown, Simon M. Barratt-Boyes

**Affiliations:** 1 Center for Vaccine Research, University of Pittsburgh, Pittsburgh, Pennsylvania, United States of America; 2 Department of Infectious Diseases and Microbiology, University of Pittsburgh, Pittsburgh, Pennsylvania, United States of America; 3 Department of Immunology, University of Pittsburgh, Pittsburgh, Pennsylvania, United States of America; SAIC-Frederick, United States of America

## Abstract

Myeloid dendritic cells (mDC) are lost from blood in individuals with human immunodeficiency virus (HIV) infection but the mechanism for this loss and its relationship to disease progression are not known. We studied the mDC response in blood and lymph nodes of simian immunodeficiency virus (SIV)-infected rhesus macaques with different disease outcomes. Early changes in blood mDC number were inversely correlated with virus load and reflective of eventual disease outcome, as animals with stable infection that remained disease-free for more than one year had average increases in blood mDC of 200% over preinfection levels at virus set-point, whereas animals that progressed rapidly to AIDS had significant loss of mDC at this time. Short term antiretroviral therapy (ART) transiently reversed mDC loss in progressor animals, whereas discontinuation of ART resulted in a 3.5-fold increase in mDC over preinfection levels only in stable animals, approaching 10-fold in some cases. Progressive SIV infection was associated with increased CCR7 expression on blood mDC and an 8-fold increase in expression of CCL19 mRNA in lymph nodes, consistent with increased mDC recruitment. Paradoxically, lymph node mDC did not accumulate in progressive infection but rather died from caspase-8-dependent apoptosis that was reduced by ART, indicating that increased recruitment is offset by increased death. Lymph node mDC from both stable and progressor animals remained responsive to exogenous stimulation with a TLR7/8 agonist. These data suggest that mDC are mobilized in SIV infection but that an increase in the CCR7-CCL19 chemokine axis associated with high virus burden in progressive infection promotes exodus of activated mDC from blood into lymph nodes where they die from apoptosis. We suggest that inflamed lymph nodes serve as a sink for mDC through recruitment, activation and death that contributes to AIDS pathogenesis.

## Author Summary

Myeloid dendritic cells (mDC) are essential innate immune system cells that are lost from blood in human immunodeficiency virus infection through an ill-defined mechanism. We studied the kinetics of the mDC response in blood and lymph nodes of rhesus macaques infected with the closely related simian immunodeficiency virus. We found that differences in the number of blood mDC correlated with eventual disease outcome, as at virus set-point mDC were increased in blood in animals remaining disease free but lost from blood in animals that progressed rapidly to AIDS. mDC loss was linked to an increase in the chemokine axis responsible for mDC recruitment to lymph nodes; however, mDC did not accumulate in tissues but rather died from apoptosis. Lymph node mDC remained responsive to stimulation with a TLR7/8 agonist during infection. Importantly, mDC dysregulation was partially reversed by antiretroviral therapy. These data indicate that chronic mDC recruitment, activation and death within lymph nodes precede development of disease in SIV infected monkeys and may play a role in AIDS pathogenesis.

## Introduction

Myeloid dendritic cells (mDC) are professional antigen-presenting cells that are critical for the induction of acquired immune responses to pathogens [1]. Depletion of mDC from blood in human immunodeficiency virus (HIV) infection has been well described and shown to be inversely correlated with virus load and absent from long-term non-progressors, suggesting a relationship between mDC and disease control [2]–[8]. A proposed mechanism to account for mDC loss from blood is their activation and subsequent recruitment to inflamed lymph nodes [9]. Increased expression of costimulatory molecules on blood mDC indicative of activation has been reported in HIV-infected individuals [3], [5], [10], as has accumulation of mDC in peripheral lymph nodes during acute infection [11]. However, findings relating to mDC in lymph nodes during chronic HIV infection are inconsistent, with both accumulation [12], [13] and substantial loss of mDC [14] being reported. mDC are depleted from both blood and lymph nodes of simian immunodeficiency virus (SIV)-infected rhesus macaques during AIDS [15] but data are lacking from earlier stages of infection. Few studies have evaluated mDC dynamics in both blood and lymph node in the same individuals [12], [15] and no longitudinal studies of mDC kinetics in both compartments have been reported. As such, the relationship between mDC loss and recruitment in infection remains ill-defined, and whether differences in mDC dynamics predict disease outcome is not known.

The impact of antiretroviral therapy (ART) on mDC loss and recovery in HIV infection is also unclear, as several studies indicate that ART is not effective at increasing blood mDC [6], [7], [16] while others suggest that ART significantly restores blood mDC numbers [3], [8], [17], [18]. ART rapidly resolves immune activation in lymphoid tissues [19] and may have beneficial effects on lymph node mDC activation and function [13], although this has not been well characterized.

In the present study we followed the mDC response in blood and lymph nodes over time in two cohorts of SIV-infected animals that received ART and adenovirus (Ad)-based immunotherapy with different disease outcomes. We find that loss of blood mDC at virus set-point is predictive of disease progression, whereas an increase in blood mDC is predictive of long-term absence of disease, and that even relatively short periods of ART are beneficial to mDC homeostasis. In animals that progress to AIDS the early loss of mDC from blood is associated with evidence of increased CCR7-CCL19-mediated recruitment to lymph nodes and increased apoptosis within these tissues.

## Results

### Disease progression is independent of Ad-based immunotherapy

Animals in this study were enrolled in an immunotherapy protocol using Ad-based vectors the majority of which has been previously described [20]. Animals were infected with the pathogenic isolate SIVmac251 by intravenous inoculation and received ART consisting of a combination of two reverse transcriptase inhibitors, 9-[2-(phosphonyl-methoxy)propyl]adenine (PMPA) and 2′-deoxy-5-fluoro-3′-thia-cytidine (FTC), from weeks 12 to 24 and weeks 32 to 44, depending on survival. Immunotherapy consisted of priming with Ad serotype 5 (Ad5)-based vectors expressing SIV Gag, Env and Nef with or without IL-15 at weeks 16 and 22 followed by boosting with Ad35-based vectors expressing the same transgenes at weeks 36 and 42. Control-treated animals were given the same regimen of Ad5-ψ5 and Ad35-ψ5 vectors that lacked transgenes [20]. Ad-based immunotherapy boosted T cell responses to SIV but had no effect on virus load, progression to disease or survival [20](and data not shown). However, when analyzed independent of immunotherapy, animals in the cohort could be readily separated into two groups based on disease progression, with one group remaining healthy until elective sacrifice at a mean of 60 weeks post infection (n = 11, ‘stable’ group), and the other succumbing to AIDS with a mean survival time of 32 weeks (n = 10, ‘progressor’ group, Table 1). AIDS was defined clinically by lymphadenopathy, persistent weight loss and anorexia, with or without opportunistic infections [15]. Equal numbers of animals in the stable and progressor groups received Ad-based immunotherapy with the remainder receiving control vectors or no treatment, confirming the lack of association between immunotherapy and disease outcome (Table 1). The MHC class I molecule Mamu-A*01 was expressed by 3/11 and 0/10 animals in the stable and progressor groups, respectively, consistent with an association of this molecule with control of SIV infection (Table 1) [21].

**Table 1:** Characteristics of animal cohort.

Animal ID	Mamu type*	Immunotherapy¶	Set point virus load (RNA copies/mL)†	Time of sacrifice (weeks PI) ‡	Disease status at sacrifice
**Stable**					
R478	A-02/A-08/B-01	Control	3,376,666	56	Healthy
R479	A-01	Ad-SIV	410,333	62	Healthy
R480	A-01	Control	796,666	56	Healthy
R481	ND	Ad-SIV/IL-15	189,333	63	Healthy
R484	A-11	Control	2,396,666	62	Healthy
R486	ND	Ad-SIV	121,666	62	Healthy
R487	A-01/B-01	Ad-SIV/IL-15	5,833,333	61	Healthy
R489	A-08/B-17	Ad-SIV	1,316,666	62	Healthy
M5306	ND	Control	99,000	63	Healthy
M5506	A-08	Ad-SIV/IL-15	3,126,666	56	Healthy
M5606	A-08	Ad-SIV/IL-15	7,300,000	60	Healthy
**Mean**			**2,269,727**	**60**	
**Progressor**					
R180	B-01	Ad-SIV	671,000	43	AIDS
R183	B-17	Ad-SIV/IL-15	706,666	43	AIDS
R189	A-02	None	47,500,000	11	AIDS
R477	ND	Ad-SIV	14,300,000	32	AIDS
R482	B-01	Control	12,733,333	27	AIDS
R483	ND	Ad-SIV	6,700,000	40	AIDS
R485	ND	Ad-SIV/IL-15	1,870,000	33	AIDS
R488	B-01	Ad-SIV/IL-15	82,000,000	18	AIDS
M5206	A-02	Ad-SIV	11,333,333	36	AIDS
M5406	ND	Control	15,700,000	42	AIDS
**Mean**			**19,351,433**	**32**	
***P***			**0.01**	**<0.0001**	

*Expression of known MHC class I Mamu alleles. ND = none of the 8 alleles tested was expressed.

**¶:** Immunotherapy was administered at weeks 16 and 22 for Ad5-based vectors and weeks 36 and 42 for Ad35-based vectors. Control = Ad-ψ5; Ad-SIV = separate vectors expressing SIV Env, Gag and Nef; Ad-SIV/IL-15 = separate vectors expressing SIV Env, Gag, Nef and IL-15.

**†:** Mean values from week 8–12 post infection.

**‡:** Animals in the stable group were electively sacrificed at or after 56 weeks post infection (PI). Animals in the progressor group were sacrificed due to development of AIDS at the indicated times.

**Figure 1:**
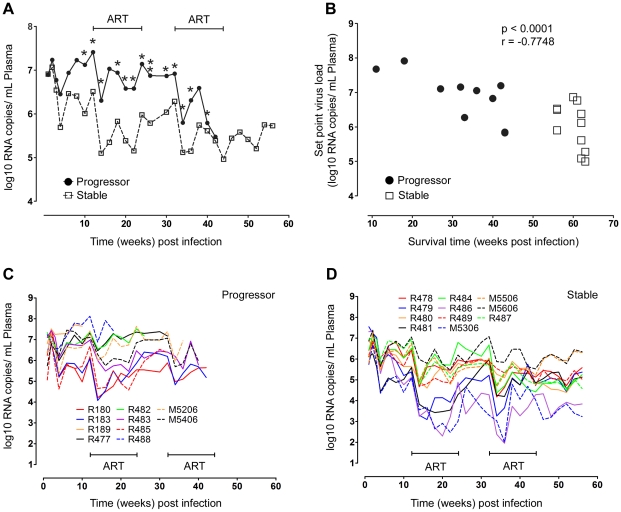
Relationship between virus load and disease progression in SIV infected macaques. (A) Mean virus load in plasma for SIV-infected animals that progress rapidly to disease (progressor, n = 10) or remain disease free for more than one year (stable, n = 11). *P<0.05, **P<0.01 (week 26). (B) Correlation between survival time and virus load at set-point, taken as the mean virus load from weeks 8 to 12 post infection, for all animals. Data points for animals R180 and R183, which died at week 43 with very similar virus loads, appear as a single data point. (C, D) Individual virus load measurements for progressor (C) and stable animals (D). ART = intervals of antiretroviral therapy.

Peak plasma virus loads in stable and progressor animals at 2 weeks post infection were similar at around 2×10^7^ RNA copies/ml plasma; however virus loads began to diverge by week 4 and at virus set point virus loads differed by ∼1 log between groups (Table 1 and Figure 1). Survival time was inversely correlated with virus load at set-point (Table 1 and Figure 1B), consistent with previous reports [22], [23]. ART beginning at week 12 had parallel although modest effects on virus load in both groups, with an immediate decrease of ∼1.5 logs that fluctuated over the course of therapy (Figure 1). Virus load persisted at ∼0.5 logs below set-point levels after discontinuation of ART and then decreased by ∼1.5 logs with initiation of the second cycle of ART at week 32, again with fluctuations over the course of therapy (Figure 1). Only animals in the stable group survived beyond the second cycle of ART and in these animals virus load remained at ∼1.5 logs below set-point until sacrifice (Table 1 and Figure 1). These data show that disease progression and survival in this cohort of animals correlated with virus load at set-point prior to initiation of ART and not with Ad-based immunotherapy, and that ART was effective at inducing modest but similar decreases in virus load in both groups.

### Disease progression correlates with a divergent mDC response in SIV-infected macaques

**Figure 2:**
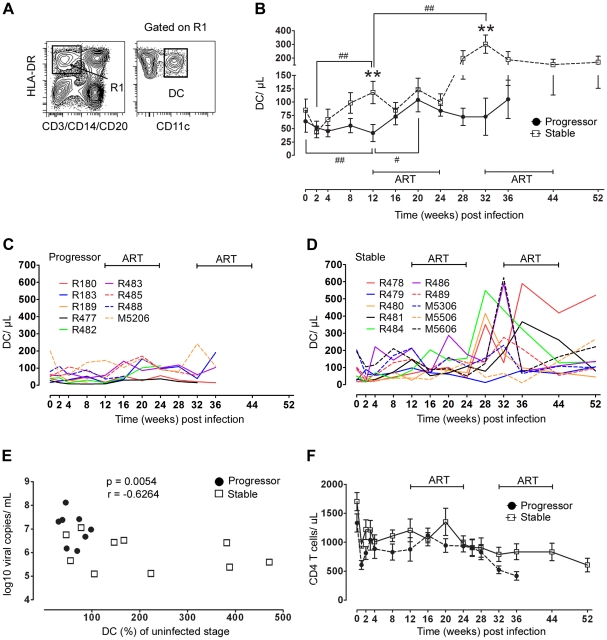
Divergent mDC response in blood correlates with virus load and disease progression. (A) Representative flow cytometry plots showing the gating strategy used to define CD11c^+^ mDC within the Lineage^−^ HLA-DR^+^ fraction of PBMC. (B) Changes in the absolute number of mDC in blood over the course of SIV infection in animals with stable (n = 10) and progressive (n = 9) infection. Shown are mean ± SEM. **P<0.01 between groups; #*P*<0.05; ##*P*<0.01 within groups relative to week 12 post infection. (C, D) Individual mDC counts in blood for progressor animals (C, n = 9) and stable animals (D, n = 10). (E) Correlation between percent change of mDC in blood from week 0 to 12 and virus load at week 12 (n = 18, R189 died at week 11 and is not included). (F) Change in absolute number of CD4 T cells in blood over the course of SIV infection in animals with stable (n = 10) and progressive (n = 9) infection. Shown are mean ± SEM. ART = intervals of antiretroviral therapy.

The characteristics of this cohort allowed us to ask whether differences in eventual disease outcome were reflected in earlier changes in the mDC response and whether short-term exposure to ART was beneficial to this response. Blood mDC were identified in peripheral blood mononuclear cells (PBMC) as CD45^+^ lineage^−^ HLA-DR^+^ CD11c^+^ cells (Figure 2A) and were enumerated based on the ratio of mDC to CD4^+^ T cells [20], [24]. Staining of blood cells with antibody to CD11c was inconsistent in animals R487 (stable group) and M5406 (progressor group) making it difficult to delineate mDC at all time points (data not shown), and as a result these animals were not studied further. The median number of blood mDC in the remaining 19 animals prior to infection was 51 cells/ul with a relatively large range from 16 to 202 cells/ul, consistent with our previous findings (Figure 2C, D) [24]. Blood mDC were reduced at 2 weeks post infection relative to baseline levels when all animals were analyzed together (*P* = .03), although when each group was analyzed separately this decrease was not significant. However, in the post-acute period the mDC response diverged, as mDC in progressor animals continued to decline to week 12 when they were significantly reduced in number relative to preinfection time points. In contrast, mDC in stable animals significantly increased from weeks 2 to 12 (Figure 2B–D). The relative change in the number of blood mDC in individual animals over the first 12 weeks of infection was significant, as mDC dropped to around 30% of preinfection levels in some progressor animals (mean for the group 60%) but increased to nearly 500% in some stable animals (mean for the group 206%) (Figure 2E). This change was inversely correlated with virus load at week 12 post infection, revealing a relationship between viral burden and mDC homeostasis (Figure 2E). Exposure to the first round of ART in progressor animals resulted in an increase in mDC number from weeks 12 to 20, when virus load was near its lowest point, and appeared to stabilize the number of blood mDC in the stable group (Figure 2B–D). However, after ART was discontinued at week 24 the number of mDC in stable but not progressor animals rose markedly reaching a mean increase of 3.5 fold over baseline at week 32, with individual increases approaching 10-fold in some animals (Figure 2B–D). The magnitude of the mDC response after ART was not influenced by the vaccination regimen received during ART, as a comparison of mean mDC counts for all animals from weeks 28 to 32 (the period of greatest response) based on the type of immunotherapy received revealed no statistically significant differences (data not shown, Kruskal-Wallis test, *P* = 0.3). Initiation of the second round of ART again reduced mDC number in the majority of stable animals, concurrent with the reduction in virus load, after which the number of mDC remained relatively constant (Figure 2B, D). The divergent mDC response contrasted with changes in CD4^+^ T cells, which did not statistically differ between groups at any time before or after infection (Figure 2F). These data indicate that differences in eventual disease outcome in SIV infection are reflected by differences in the blood mDC response that are apparent relatively early in infection. They also indicate that short-term ART may be effective at transiently restoring blood mDC in animals with the most severe disease.

### Blood mDC are rapidly and differentially activated in SIV infection

**Figure 3:**
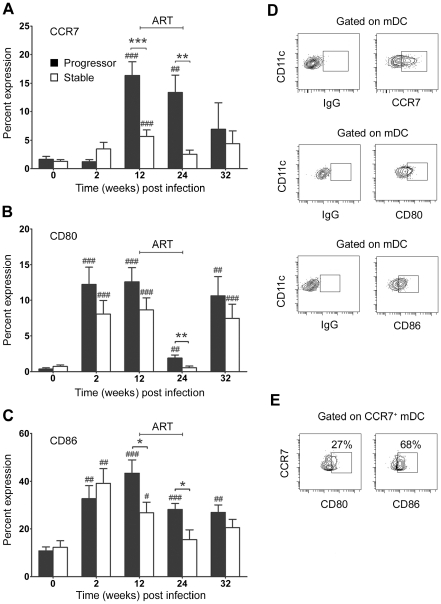
Differential activation of blood mDC in stable and progressive SIV infection. The proportion of blood mDC expressing CCR7 (A), CD80 (B) and CD86 (C) relative to staining with a control antibody before and at various times after SIV infection. Shown are mean ± SEM for naïve (n = 19), progressor (n = 8) and stable animals (n = 10). (D) Representative flow cytometry plots of the gating strategy to identify positive populations. (E) Flow cytometry plots of CCR7^+^ mDC from progressor animal R180 at 12 weeks post infection labeled with antibodies to CD80 and CD86. Numbers represent the percentage of cells that co-express both markers relative to staining with a control antibody. Data are representative of three experiments on separate animals. *P<0.05, **P<0.01, ***P<0.005 between groups; #*P*<0.05, ##*P*<0.01, ###*P*<0.005 within groups relative to week 0. ART = interval of antiretroviral therapy.

We next asked whether differences in disease progression were reflected in earlier differences in activation of circulating mDC in SIV-infected macaques. For these and subsequent analyses we focused on the first 32 weeks of infection incorporating one 12-week cycle of ART and one 8-week period of treatment interruption, as after this time the number of animals surviving in the progressor group rapidly diminished (Table 1). Expression of the costimulatory molecules CD80 and CD86 was markedly increased in all animals at 2 weeks post infection indicative of rapid mDC activation (Figure 3A–D). However, by 12 weeks post infection differences in mDC activation were evident between groups particularly with respect to the chemokine receptor CCR7, which was expressed by a significantly greater proportion of mDC in animals that progressed to AIDS relative to animals with stable infection (Figure 3A). A majority of CCR7^+^ mDC in progressor animals also expressed CD86 with a smaller proportion expressing CD80, consistent with activation (Figure 3E). The 12-week course ART was effective at reducing blood mDC activation, particularly with respect to CD80, and in animals in the stable group expression of all markers of activation returned to preinfection levels during ART (Figure 3A–C). The increase in CD80 at week 32 suggested mDC were again activated during the period of ART discontinuation, although no increase in CCR7 or CD86 expression was noted at this time (Figure 3A–C). These findings indicate that during chronic infection animals that progress to AIDS have increased blood mDC activation relative to animals with stable infection. They also confirm that ART has the beneficial effect of reducing mDC activation, consistent with findings in HIV-infected humans [3], [12].

### mDC do not accumulate in lymph nodes despite increased tissue CCL19 expression

**Figure 4:**
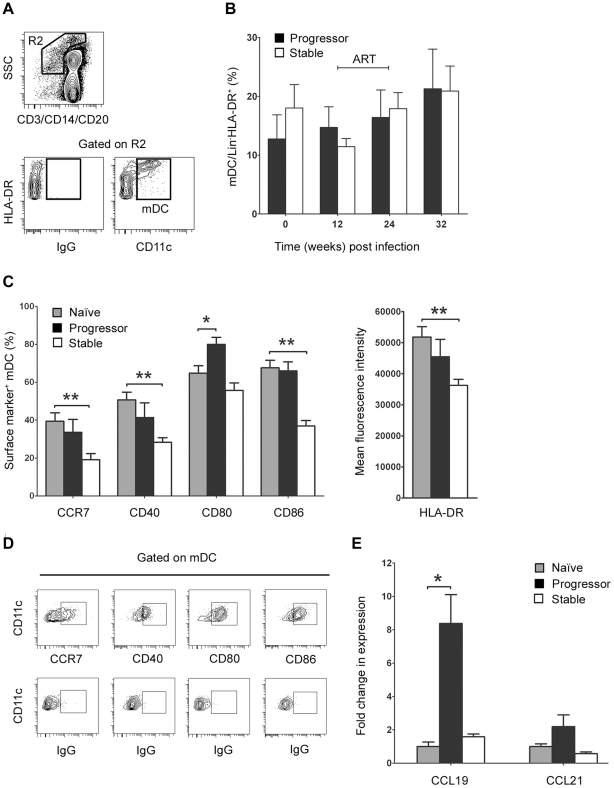
Differential mDC activation and tissue chemokine expression in lymph nodes correlates with disease progression. (A) Representative flow cytometry plots showing the gating strategy used to define CD11c^+^ mDC within the Lineage^−^ HLA-DR^+^ fraction of peripheral lymph node cell suspensions. (B) The number of mDC in lymph nodes as a proportion of Lineage^−^ HLA-DR^+^ cells at intervals before and after SIV infection. Shown are mean ± SEM for naïve (T = 0, n = 18), progressor (n = 7) and stable animals (n = 10). (C) The proportion of lymph node mDC expressing CCR7, CD40, CD80 and CD86 relative to staining with a control antibody before and 12 weeks after SIV infection (left) and the mean fluorescence intensity of HLA-DR expression on lymph node mDC at week 12 post infection (right). Shown are mean ± SEM for naïve (n = 8), progressor (n = 6) and stable animals (n = 6). (D) Representative flow cytometry plots showing gating strategy to identify positive cell populations. (E) Expression of CCL19 and CCL21 mRNA in lymph node cell suspensions prior to infection (naïve) and at 12 weeks post infection for animals with stable and progressive infection. mRNA expression levels were calculated using the 2^−Δ^C_T_ method using [beta]-GUS as the endogenous control. The fold change in expression was calculated by normalizing to the mean 2^−Δ^C_T_ of the naïve group. Shown are mean ± SEM of 4 animals in each group. *P<0.05, **P<0.01. ART = interval of antiretroviral therapy.

The finding that loss of blood mDC in progressor animals occurs as the proportion of mDC expressing CCR7 increases is consistent with excessive mDC recruitment to lymph nodes via the CCR7/CCL19/CCL21 pathway, as has been suggested by in vitro studies [9]. To examine this potential in vivo, we used flow cytometry to identify mDC in lymph node cell suspensions taken prior to infection and at intervals after infection in our two groups of animals. mDC were defined as lineage^−^ HLA-DR^+^ CD11c^+^ cells (Figure 4A) and enumerated as a proportion of all cells in the lineage^−^ HLA-DR^+^ gate, which we have previously shown to be an accurate indicator of the absolute number of mDC per unit of weight [15]. Surprisingly, we found no significant difference in the number of lymph node mDC as a result of SIV infection regardless of disease progression, indicating a lack of mDC accumulation (Figure 4B). However, the phenotype of mDC within lymph nodes was significantly different as a function of disease, as animals with stable but not progressive infection had a lower percentage of mDC expressing CCR7, CD40 and CD86 and reduced mDC expression of MHC class II at 12 weeks relative to preinfection time points, reflecting reduced mDC activation (Figure 4C, D). To address the issue of mDC recruitment further, we next used real time PCR to determine the relative expression of CCR7 ligands in lymph node tissues. We found that CCL19 but not CCL21 mRNA was increased 8-fold in lymph nodes at 12 weeks post infection, but only in animals that progressed to AIDS (Figure 4E). Together with our findings in blood, these data suggest that mDC are recruited to lymph nodes in progressive disease via an enhanced CCR7/CCL19 pathway, but that expanded mDC recruitment fails to result in mDC accumulation.

### Increased mDC apoptosis in lymph nodes during progressive SIV infection

**Figure 5:**
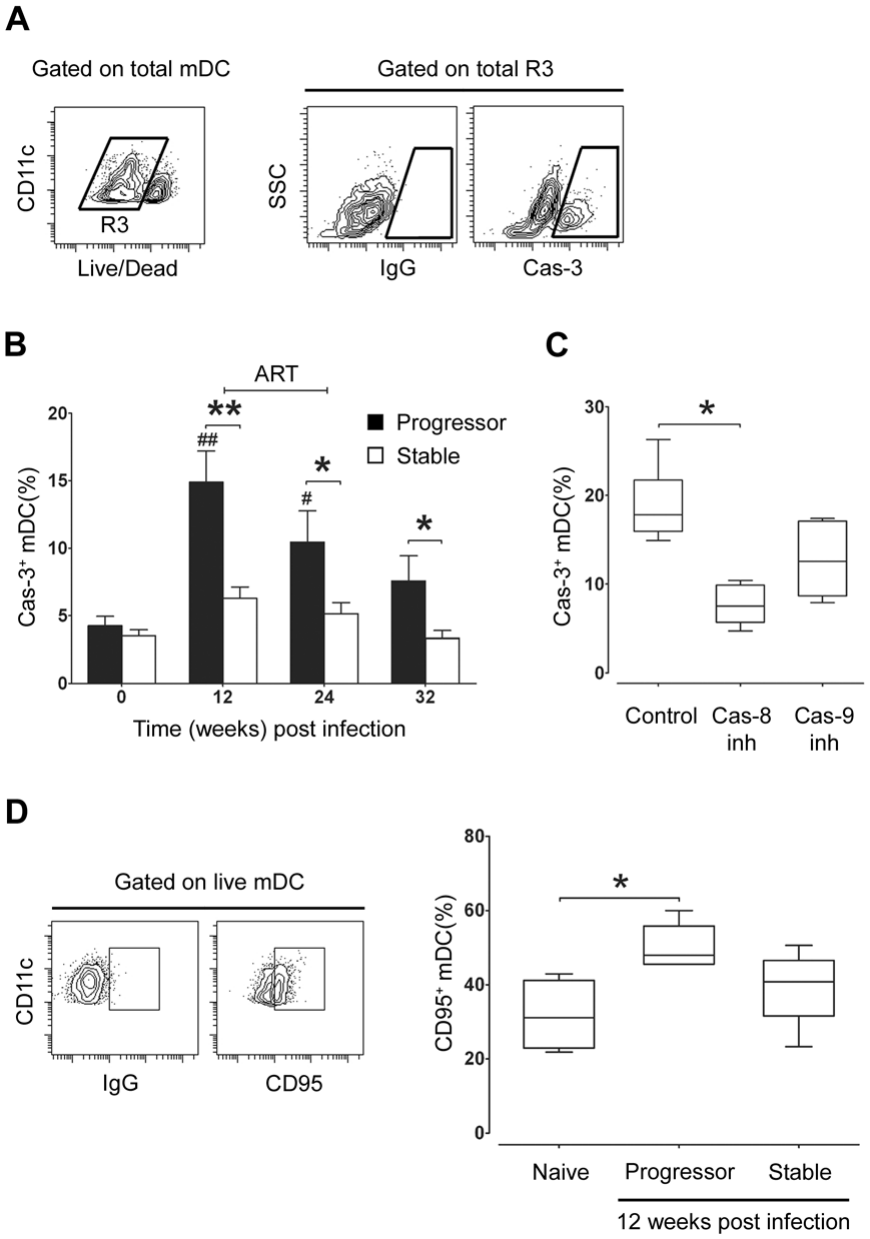
Apoptosis of lymph node mDC increases in progressive but not stable SIV infection. (A) Representative flow cytometry plots showing the gating strategy used to identify mDC expressing active caspase-3 (Cas-3). Live mDC were defined as Lineage^−^ HLA-DR^+^ CD11c^+^ cells that lacked staining with a fixable dead-cell dye. (B) The proportion of lymph node mDC expressing active caspase-3 relative to staining with a control antibody at intervals before and after SIV infection. Cells were cultured for 3 hours prior to analysis. Shown are mean ± SEM for naïve (T = 0, n = 14), progressor (n = 8) and stable n = 10). (C) The proportion of lymph node mDC, taken at 12 weeks post infection from animals with progressive infection, expressing active caspase-3 after 3 hours in the presence of caspase-8 inhibitor (Cas-8 inh), caspase-9 inhibitor (Cas-9 inh) or control inhibitor (Control). Boxes represent 25^th^ to 75^th^ percentile and median values, and whiskers represent the minimum and maximum values, using data from 6 animals. (D) Left: Representative dot plots showing expression of CD95 relative to isotype control antibody to define positively staining cells. Right: The proportion of lymph node mDC at 12 weeks post infection that express CD95 in naïve animals (n = 4) and animals with progressive (n = 5) and stable infection (n = 5). Boxes represent 25^th^ to 75^th^ percentile and median values, and whiskers represent minimum and maximum values. *P<0.05, **P<0.01 between groups; #*P*<0.05, ##*P*<0.01, within groups relative to week 0. ART = intervals of antiretroviral therapy.

The lack of mDC accumulation in lymph nodes despite evidence for enhanced CCR7/CCL19-mediated recruitment in progressive infection led us to suspect that lymph node mDC were dying at an increased rate in these tissues. To examine this possibility we identified live mDC in lymph node cell suspensions as being lineage^−^ HLA-DR^+^ CD11c^+^ cells that lacked staining with a fixable dead-cell dye, and then identified cells undergoing early apoptosis within this gate using an antibody to active caspase-3 (Figure 5A). At week 12 post infection, 15% of lymph node mDC in animals that eventually progressed to AIDS were entering apoptosis, representing a 3-fold increase from preinfection levels, whereas lymph node mDC from animals with stable infection had no significant change in apoptosis (Figure 5B). ART given from week 12 to 24 post infection decreased the frequency of apoptotic mDC in progressor animals, although this did not reach statistical significance (Figure 5B). To determine whether apoptosis was mediated by extrinsic or intrinsic pathways we exposed lymph node cells from progressor animals taken at week 12 post infection to small molecule inhibitors of caspase-8 or caspase-9, respectively. The presence of caspase-8 inhibitor Z-IETD-FMK reduced apoptosis by more than 50% relative to a control peptide whereas the caspase-9 inhibitor had minimal effect (Figure 5C), suggesting that cell-extrinsic mediators of apoptosis were predominant. Consistent with this finding, lymph node mDC taken from progressor but not stable animals at week 12 post infection showed a significant increase in the proportion of mDC expressing CD95 relative to preinfection samples (Figure 5D). Together, these data suggest that increased mDC apoptosis in lymph nodes during chronic infection in animals that progress to AIDS offsets the increase in mDC recruitment from blood, resulting in no net accumulation of mDC.

### Evidence of increased responsiveness of lymph node mDC to stimulation with TLR7/8 agonist during infection

**Figure 6:**
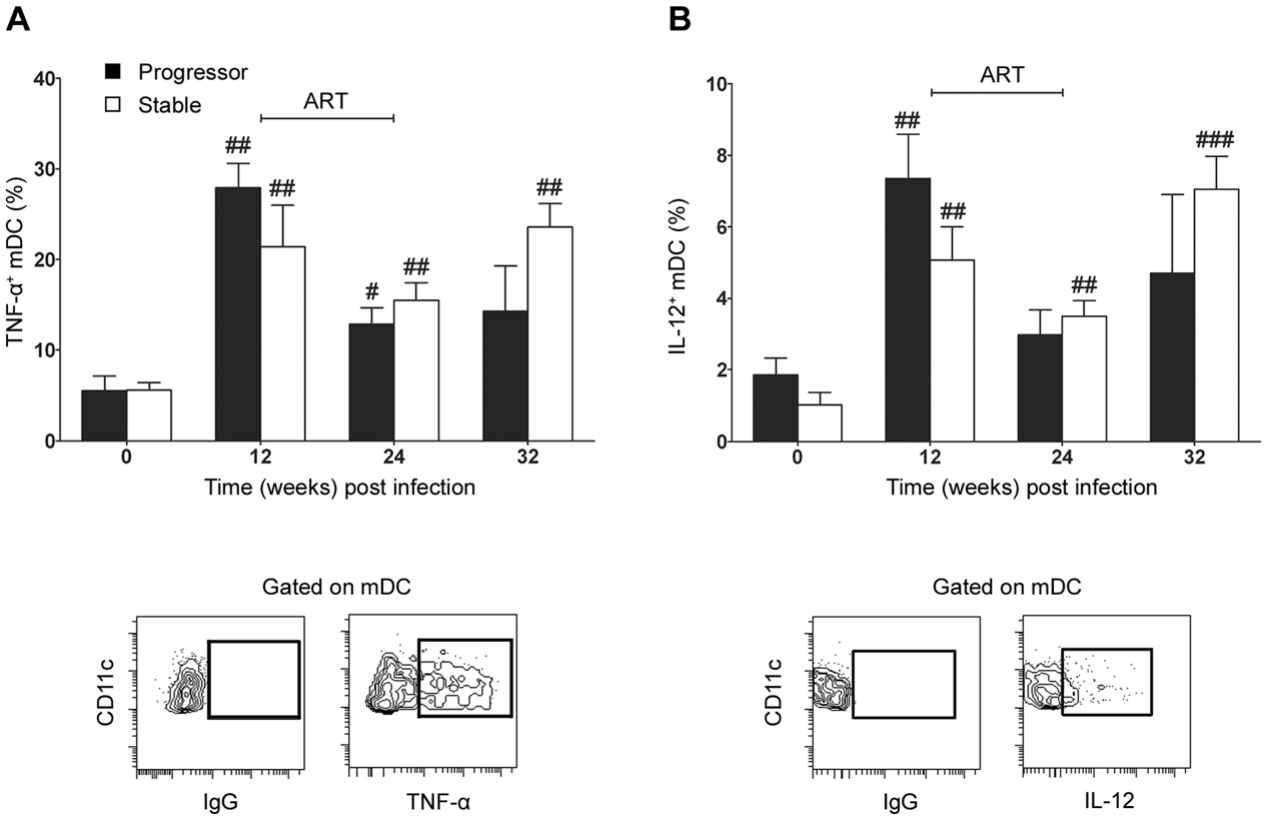
Lymph node mDC from SIV infected animals remain responsive to exogenous TLR8 stimulation. The proportion of lymph node mDC before and after SIV infection that express (A) TNF-α (A) or IL-12 (B) following stimulation with the TLR7/8 agonist 3M-007 for 5 hours. Shown are mean ± SEM for naïve (T = 0, n = 13), progressor (n = 7) and stable animals (n = 10). Also shown are representative flow cytometry plots of the gating strategy to identify positive populations. #*P*<0.05, ##*P*<0.01 within groups relative to week 0. ART = interval of antiretroviral therapy.

Changes in mDC activation and apoptosis within lymph nodes during SIV infection could impact the capacity of these cells to respond to microbial stimuli and subsequently induce adaptive T cell immune responses. To investigate the functional capacity of mDC following SIV infection in our two groups of animals we stimulated lymph node cell suspensions taken at intervals before and after infection with 3M-007, a small molecule synthetic agonist of TLR7/8, which, like HIV and SIV RNA, activates mDC through their engagement of TLR8 [25]–[27]. We analyzed mDC for expression of two key immunoregulatory cytokines, TNF-α and IL-12 (p40/p70). Interestingly, lymph node mDC taken prior to infection responded relatively poorly to short-term stimulation with a small proportion of cells producing TNF-α and IL-12 (Figure 6). In contrast, stimulation of mDC taken at 12 weeks post infection resulted in 20 to 30% of cells producing TNF-α and a smaller but significant percentage producing IL-12, representing a 4- to 5-fold increase above preinfection levels regardless of disease outcome (Figure 6). ART reduced mDC responsiveness to TLR8 stimulation although this did not reach statistical significance (Figure 6B). These data indicate that mDC resident in lymph nodes of SIV-infected rhesus macaques are functional capable of responding to stimulation, and may even be hyperresponsive as a consequence of SIV infection.

## Discussion

In this study we examined the relationship between mDC dynamics and disease progression over time in pathogenic SIV infection of rhesus macaques. We show for the first time that mDC are preferentially lost from blood in animals that progress to AIDS but are increased in blood of animals with long-term stable infection. This divergent mDC response was apparent at virus set-point, indicating that changes in blood mDC number over the first 3 months of infection are predictive of eventual disease progression.

mDC are recruited from blood to lymphoid tissues through upregulation of CCR7, the ligand for chemokines CCL19 and CCL21 that are expressed in the lymph node paracortex [28]. In animals with progressive infection, mDC loss from blood was associated with an increase in the frequency of blood mDC expressing CCR7 and an increase in expression of CCL19 in lymph nodes, consistent with increased extravasation to lymph nodes that exceeded the rate of mDC production from bone marrow. Expression of CCL19 has been shown previously to be markedly increased in lymph nodes during the acute phase of SIV infection [29], and our data suggest that expression in lymph nodes remains high into chronic infection as a function of virus load. Indeed, recent studies have shown that increased levels of CCL19 and CCL21 in blood correlate with higher virus loads and disease progression in HIV infected humans [30]. In vitro exposure to CCL19 and CCL21 also promotes an inflammatory response in PBMC from HIV-infected individuals with high virus loads [31]. We now provide evidence of a functional link between CCL19 upregulation in lymph nodes and increased expression of CCR7 on circulating mDC that promotes mDC recruitment to lymph nodes in progressive SIV infection. While not examined in this study, the potential exists for proinflammatory factors to promote differential emigration of mDC to lymph nodes in progressive relative to stable SIV infection. In particular, lipopolysaccharide is increased in the circulation during chronic HIV and pathogenic SIV infection as a consequence of microbial translocation through increased gut permeability [32]. Lipopolysaccharide activates mDC via engagement of TLR4 [33] and is a potent activator of DC migration in vivo [34], [35].

In contrast to progressive infection, we found that mDC in animals that controlled SIV infection had significant increases in blood mDC over time, with increases of up to 5-fold by virus set-point and nearly 10-fold in some cases at 32 weeks of infection. Studies in HIV infected individuals have indicated that mDC loss is inversely proportional to virus load, as we have shown, and is not observed in long-term non-progressors [2], [5], [8], but such cross sectional studies have by design not revealed changes over time. Increased blood mDC may arise from increased hematopoiesis in bone marrow in response to inflammatory cytokines such as TNF-α and IL-1 that are elevated during HIV infection and promote DC generation [36], [37]. The lack of an upregulated CCR7-CCL19 axis in this group would exacerbate the impact of enhanced DC production and mobilization into blood by limiting mDC exodus into tissues.

Paradoxically, there was no net increase in mDC within lymph nodes in monkeys with progressive SIV infection, associated with an increase in mDC apoptosis, suggesting that increased recruitment to lymph nodes is offset by increased cell death in severe infection. mDC apoptosis was caspase-8-dependent and associated with increased CD95 expression, similar to the findings for plasmacytoid DC in HIV and SIV infection [38], [39], consistent with a cell-extrinsic mechanism of apoptosis involving CD95 ligation. Apoptosis through virus infection of mDC is unlikely to be a significant factor, as previous studies indicate that only a minor fraction of lymph node mDC contain incorporated viral DNA during peak viremia [38]. HIV and SIV clearly affect mDC in the absence of productive infection, in particular through interactions of viral RNA with endosomal TLR8; however this interaction tends to promote cell survival rather than apoptosis [9], [27]. While the increase in mDC recruitment appears to keep pace with apoptosis in tissues during the chronic stages of SIV infection studied here it is clear that mDC are ultimately lost from lymph nodes as AIDS is established, as previously reported [15]. This eventual decline may be associated with a similar decline in lymph node expression of CCL19 in the final stages of disease [29].

Several reports have described the presence of semimature mDC with reduced expression of costimulatory molecules and/or CD83 in lymph node and spleen of HIV-infected humans [11], [13], [40] and SIV-infected macaques [41], [42]. Our data now suggest that these cells may have a beneficial function in vivo, as lymph node mDC with significantly lower expression of CCR7 and costimulatory molecules consistent with a semimature state were present only in animals with long-term stable infection. In vitro, semimature DC with tolerogenic function are derived from exposure to immunoregulatory cytokines including IL-10 and transforming growth factor-β [43], however whether these factors modulate DC maturation and function in progressive versus stable SIV and HIV infection is not known. Semimature mDC from HIV-infected lymph nodes have been shown to promote regulatory T cell function [13]. While we were not able to examine the effect of these cells on regulatory T cells in this study, the prevalence of semimature mDC in stable but not progressive infection might suggest a role for enhanced regulatory T cell responses in disease control. The role of regulatory T cells in pathogenic and nonpathogenic SIV infection is currently controversial [44]–[46], and the interplay between mDC and regulatory T cells in control and progression to disease deserves attention. In contrast to stable infection, mDC in lymph nodes of animals with progressive infection showed essentially no difference in expression of CCR7 and activation markers relative to naïve animals, although the proportion of cells expressing these markers was substantially greater than in blood. It is possible that activated mDC undergo apoptosis immediately upon entering lymph nodes, or alternatively that other newly identified costimulatory molecules from the CD28 and TNFR families not examined here may be differentially expressed in progressively infected lymph nodes [47].

In our study the two short courses of ART had only modest although similar effects on virus load in both groups of animals, reducing virus levels in plasma by ∼1.5 logs. This may be due to the fact that ART was initiated in chronic as opposed to acute infection and that therapy was limited to PMPA and FTC which both target the same viral protein, reverse transcriptase. Similarly limited effects of ART on virus load in SIV infection have been reported by others [48], [49]. Despite this, ART had noticeably beneficial effects on mDC homeostasis. In blood, ART reduced mDC activation and transiently restored mDC numbers in monkeys with progressive infection, consistent with reports in HIV-infected individuals [8], [17], [18]. Most strikingly, discontinuation of ART in stable animals led to a marked increase in the number of mDC in blood. In lymph nodes, ART resulted in a decrease in mDC apoptosis in animals with progressive infection and a reduction in mDC responsiveness overall. Consistent with this finding, expression of proinflammatory factors and CD95L that likely induce functional activation and apoptosis of mDC are substantially reduced in SIV- and HIV-infected lymph nodes in response to ART [19], [50], [51]. Animals in the progressive infection group died at a median time of 34 weeks post infection and did not receive the full second course of ART initiated at week 32. We do not believe this difference in treatment interval was a determining factor in survival, as disease status and time to sacrifice were correlated with virus load at set-point, before initiation of any therapy, and thus were independent of ART. HIV-infected individuals with higher baseline virus loads and immune activation have poorer reconstitution of innate immune cells in response to ART [16], [52]. In our study, differences in baseline virus load influenced the response to ART, as animals with stable and progressive infection had transient increases and decreases, respectively, in the number of blood mDC, although this could clearly be influenced by the differences in virus load in the two groups while on ART. It will be important to determine the impact of improved antiretroviral drug regimens on mDC dynamics in SIV infection, including the orally available integrase inhibitors that are highly active in monkeys [53].

Our data indicate that mDC present in lymph nodes in SIV infected monkeys remain functionally responsive to exogenous stimulation regardless of disease outcome. The finding that ex vivo stimulation through TLR8 induced a five-fold increase in expression of TNF-α relative to naïve animals suggests that these cells may in fact be hyperresponsive, although testing with a more extensive panel of agonists targeting different TLR ligands is needed to confirm this. CCL19 induces terminal activation of DC and promotes DC production of proinflammatory cytokines within lymph nodes [54], although this effect would not explain the finding of increased responsiveness of mDC in animals with stable infection that had normal levels of CCL19 in our study. It is possible that other proinflammatory factors such as IFN-γ that induce DC activation [55] and are markedly increased in lymph nodes during pathogenic SIV infection [56] are responsible for mDC increased responsiveness in SIV infection.

An increasing emphasis in HIV and SIV pathogenesis is now placed on the role of gut mucosa in disease, as this is a major site of virus replication and CD4^+^ T cell depletion [57]–[59]. mDC are recruited to inflamed respiratory mucosal surfaces in children with respiratory viral infections [60], and it is likely mDC and other DC subsets are similarly recruited to gut and vaginal mucosa in SIV infection [61]. It will be important to evaluate the mDC response in gut mucosa and its relationship to disease progression in SIV infection. However, such quantitative studies are technically difficult to perform as the DC is a relatively rare cell that can only be isolated in sufficient numbers through gut resection surgeries as opposed to the more commonly performed endoscopic biopsies.

Collectively, these data suggest that the inflammatory response associated with increased virus load during progressive SIV infection leads to an increase in the CCR7-CCL19 chemokine axis that serves to accelerate mDC recruitment to lymph nodes. Apoptosis of mDC within tissues during this chronic phase, which was found only in animals with progressive infection, would compromise the innate and adaptive immune response to opportunistic pathogens promoting disease progression. It is currently not clear whether recently recruited and activated mDC produce increased levels of proinflammatory cytokines in vivo that may mediate immune activation characteristic of HIV and pathogenic SIV infection [62]. Interestingly, increased turnover of blood monocytes associated with apoptosis of tissue macrophages has been shown to correlate with progression to disease in SIV-infected macaques and is a better predictive marker than viral load or lymphocyte activation [63], [64]. This response is not likely limited to lymph nodes, as evidenced by the fact that increased monocyte turnover and recruitment to brain correlates with the severity of SIV encephalitis [65]. These findings point to a broad-based dysregulation of mDC and monocytes in blood and tissues as a significant factor in the pathogenesis of AIDS.

## Materials and Methods

### Ethics statement

This study was carried out in strict accordance with the recommendations in the Guide for the Care and Use of Laboratory Animals of the National Institutes of Health. The protocol was approved by the Institutional Animal Care and Use Committee at the University of Pittsburgh (Assurance Number A3187-01). Surgeries were performed under anesthesia induced and maintained with ketamine hydrochloride and medetomidine hydrochloride, and all efforts were made to minimize suffering.

### Animal manipulations

Twenty one Indian-origin rhesus macaques (*Macaca mulatta*) used in this study were housed at the University of Pittsburgh Primate Facility for Infectious Disease Research. All animals were infected by intravenous inoculation with 1,000 TCID_50_ of uncloned, pathogenic SIVmac251 (provided by Christopher J. Miller, California National Primate Research Center). Virus load in plasma was determined as described previously [66]. ART consisted of PMPA (20 mg/kg/d, subcutaneous injection) and FTC (30 mg/kg/d, subcutaneous injection; both provided by Michael Miller, Gilead Sciences) and was given from weeks 12–24 and from weeks 32–44 as described [20]. All animals except R189 (sacrificed at week 11 post infection) received one or more administrations of Ad-based vectors during the study depending on survival. Priming injections of separate Ad5-based vectors expressing codon-optimized SIVmac239 *gag*, *env* and *nef* with and without rhesus *IL-15.FLAG* or empty Ad-ψ5 were given by intramuscular injection at week 16 (5×10^10^ total viral particles) and week 22 (1×10^11^ total viral particles), and boosting injections of the same quantity of Ad35-based vectors expressing the same transgenes were given at week 36 and 42. All Ad vectors were E1/E3-deleted with the exception of Ad35 containing the *env* gene which was E3 deleted. Lymph nodes were taken from the axillary or inguinal regions prior to infection and at weeks 12, 24 and 32 post infection and single cell suspensions were generated by disruption and digestion with collagenase D, as described [67].

### Cell identification and enumeration

Identification of mDC was performed as previously described with some modifications [15], [24]. Briefly, PBMC or lymph node cell suspensions were stained with fluorescently-labeled antibodies to Lineage markers [CD3 (clone SP34-2; all antibodies from BD Bioscience unless otherwise noted), CD14 (M5E2), and CD20 (2H7)], HLA-DR (G46-6) and CD11c (S-HCL-3), with and without antibodies to CD45 (D058-1283), CD80 (L307.4), CD86 (FUN-1), CCR7 (150503, R&D Systems), CD40 (5C3) and CD95 (DX2). An amine-reactive fixable dead-cell dye (Invitrogen) was used to discriminate live from dead cells. mDC were defined as Lineage^−^ HLA-DR^+^ cells expressing CD11c. In lymph nodes a broad Lineage^−^ HLA-DR^+/++^ gate was used to include all mDC as described previously The number of blood CD4^+^ T cells was quantified using a precise volume of blood stained with antibodies in the absence of any wash step in TruCOUNT tubes (BD Biosciences) that contained a known number of fluorescent beads to provide internal calibration, as previously reported [20]. The number of blood mDC was then calculated based on the ratio of mDC to CD4^+^ T cells in PBMCs at the same time point [24]. All analyses were done on an LSR II flow cytometer with FACSDiva software (BD Bioscience).

### Cytokine expression and apoptosis

Intracellular cytokine production by lymph node mDC was measured as described previously for plasmacytoid DC with minor modifications [38]. Briefly, cell suspensions were cultured for 5 hours with 10 µM of the TLR7/8 agonist 3M-007 (3 M Pharmaceuticals) with and without the addition of 10 µg/mL brefeldin A (Sigma) after 1 hour. Cells were stained with surface-labeling antibodies as above and fixed and permeabilized prior to incubation with antibody to TNF-α (MAb11) and IL-12 (8.6, Mitenyi Biotec) and analysis by flow cytometry. To detect apoptosis in mDC, lymph node cell suspensions were cultured in media for 3 hours with and without caspase-8 inhibitor Z-IETD-FMK, caspase-9 inhibitor Z-LEHD-FMK or irrelevant peptide Z-Fa-FMK (BD Biosciences). Cells were stained with surface-labeling antibodies as above and fixed and permeabilized prior to incubation with antibody to active caspase-3 (C92-605) and analysis by flow cytometry.

### Detection of chemokine mRNA expression

Total lymph node RNA was extracted and purified from cell suspensions generated from biopsies taken prior to or 12 weeks after infection using the RNAeasy kit (Qiagen) after treatment with DNAse I (Invitrogen). cDNA was synthesized using random primers and Superscript II reverse transcriptase (Invitrogen). Primers and probes from Taqman human gene expression arrays (Applied Biosystems, Foster City, CA) were utilized for real time PCR analysis of CCL19, CCL21 and β-glucuronidase expression as previously described [68]. mRNA expression levels for each gene were calculated with the 2^−Δ^C_T_ method using β-glucuronidase as the endogenous control [69].

### Statistical analysis

Comparisons between two groups were carried out using the Mann-Whitney U test. Comparison of DC numbers across different time points was carried out using the Wilcoxon signed-rank test. Correlations were determined using the non-parametric Spearman rank test. Graphpad Prism 5 (Graphpad Software) was used for statistical analysis. All *P* values are two-sided with significance considered to be *P*<0.05.

### Gene identification

The identification of genes analyzed in this paper as defined by Entrez-Gene are 574386 (CCL19), 574183 (CCL21) and 677692 (β-glucuronidase).

## References

[1] Shortman K, Liu YJ (2002). Mouse and human dendritic cell subtypes.. Nat Rev Immunol.

[2] Almeida M, Cordero M, Almeida J, Orfao A (2005). Different subsets of peripheral blood dendritic cells show distinct phenotypic and functional abnormalities in HIV-1 infection.. AIDS.

[3] Barron MA, Blyveis N, Palmer BE, MaWhinney S, Wilson CC (2003). Influence of plasma viremia on defects in number and immunophenotype of blood dendritic cell subsets in human immunodeficiency virus 1-infected individuals.. J Infect Dis.

[4] Donaghy H, Pozniak A, Gazzard B, Qazi N, Gilmour J (2001). Loss of blood CD11c(+) myeloid and CD11c(−) plasmacytoid dendritic cells in patients with HIV-1 infection correlates with HIV-1 RNA virus load.. Blood.

[5] Grassi F, Hosmalin A, McIlroy D, Calvez V, Debre P (1999). Depletion in blood CD11c-positive dendritic cells from HIV-infected patients.. AIDS.

[6] Pacanowski J, Kahi S, Baillet M, Lebon P, Deveau C (2001). Reduced blood CD123+ (lymphoid) and CD11c+ (myeloid) dendritic cell numbers in primary HIV-1 infection.. Blood.

[7] Fontaine J, Coutlee F, Tremblay C, Routy JP, Poudrier J (2009). HIV infection affects blood myeloid dendritic cells after successful therapy and despite nonprogressing clinical disease.. J Infect Dis.

[8] Chehimi J, Campbell DE, Azzoni L, Bacheller D, Papasavvas E (2002). Persistent decreases in blood plasmacytoid dendritic cell number and function despite effective highly active antiretroviral therapy and increased blood myeloid dendritic cells in HIV-infected individuals.. J Immunol.

[9] Fonteneau JF, Larsson M, Beignon AS, McKenna K, Dasilva I (2004). Human immunodeficiency virus type 1 activates plasmacytoid dendritic cells and concomitantly induces the bystander maturation of myeloid dendritic cells.. J Virol.

[10] Jones GJ, Watera C, Patterson S, Rutebemberwa A, Kaleebu P (2001). Comparative loss and maturation of peripheral blood dendritic cell subpopulations in African and non-African HIV-1-infected patients.. Aids.

[11] Lore K, Sonnerborg A, Brostrom C, Goh LE, Perrin L (2002). Accumulation of DC-SIGN+CD40+ dendritic cells with reduced CD80 and CD86 expression in lymphoid tissue during acute HIV-1 infection.. Aids.

[12] Dillon SM, Robertson KB, Pan SC, Mawhinney S, Meditz AL (2008). Plasmacytoid and myeloid dendritic cells with a partial activation phenotype accumulate in lymphoid tissue during asymptomatic chronic HIV-1 infection.. J Acquir Immune Defic Syndr.

[13] Krathwohl MD, Schacker TW, Anderson JL (2006). Abnormal presence of semimature dendritic cells that induce regulatory T cells in HIV-infected subjects.. J Infect Dis.

[14] Biancotto A, Grivel JC, Iglehart SJ, Vanpouille C, Lisco A (2007). Abnormal activation and cytokine spectra in lymph nodes of people chronically infected with HIV-1.. Blood.

[15] Brown KN, Trichel A, Barratt-Boyes SM (2007). Parallel loss of myeloid and plasmacytoid dendritic cells from blood and lymphoid tissue in simian AIDS.. J Immunol.

[16] Azzoni L, Chehimi J, Zhou L, Foulkes AS, June R (2007). Early and delayed benefits of HIV-1 suppression: timeline of recovery of innate immunity effector cells.. Aids.

[17] Finke JS, Shodell M, Shah K, Siegal FP, Steinman RM (2004). Dendritic cell numbers in the blood of HIV-1 infected patients before and after changes in antiretroviral therapy.. J Clin Immunol.

[18] Almeida M, Cordero M, Almeida J, Orfao A (2006). Persistent abnormalities in peripheral blood dendritic cells and monocytes from HIV-1-positive patients after 1 year of antiretroviral therapy.. J Acquir Immune Defic Syndr.

[19] Li Q, Schacker T, Carlis J, Beilman G, Nguyen P (2004). Functional genomic analysis of the response of HIV-1-infected lymphatic tissue to antiretroviral therapy.. J Infect Dis.

[20] Soloff AC, Liu X, Gao W, Day RD, Gambotto A (2009). Adenovirus 5- and 35-based immunotherapy enhances the strength but not breadth or quality of immunity during chronic SIV infection.. Eur J Immunol.

[21] Mothe BR, Weinfurter J, Wang C, Rehrauer W, Wilson N (2003). Expression of the major histocompatibility complex class I molecule Mamu-A*01 is associated with control of simian immunodeficiency virus SIVmac239 replication.. J Virol.

[22] Watson A, Ranchalis J, Travis B, McClure J, Sutton W (1997). Plasma viremia in macaques infected with simian immunodeficiency virus: plasma viral load early in infection predicts survival.. J Virol.

[23] Staprans SI, Dailey PJ, Rosenthal A, Horton C, Grant RM (1999). Simian immunodeficiency virus disease course is predicted by the extent of virus replication during primary infection.. J Virol.

[24] Brown KN, Barratt-Boyes SM (2009). Surface phenotype and rapid quantification of blood dendritic cell subsets in the rhesus macaque.. J Med Primatol.

[25] Beignon AS, McKenna K, Skoberne M, Manches O, DaSilva I (2005). Endocytosis of HIV-1 activates plasmacytoid dendritic cells via Toll-like receptor-viral RNA interactions.. J Clin Invest.

[26] Mandl JN, Barry AP, Vanderford TH, Kozyr N, Chavan R (2008). Divergent TLR7 and TLR9 signaling and type I interferon production distinguish pathogenic and nonpathogenic AIDS virus infections.. Nat Med.

[27] Meier A, Alter G, Frahm N, Sidhu H, Li B (2007). MyD88-dependent immune activation mediated by human immunodeficiency virus type 1-encoded Toll-like receptor ligands.. J Virol.

[28] Forster R, Davalos-Misslitz AC, Rot A (2008). CCR7 and its ligands: balancing immunity and tolerance.. Nat Rev Immunol.

[29] Choi YK, Fallert BA, Murphey-Corb MA, Reinhart TA (2003). Simian immunodeficiency virus dramatically alters expression of homeostatic chemokines and dendritic cell markers during infection in vivo.. Blood.

[30] Damas JK, Landro L, Fevang B, Heggelund L, Froland SS (2009). Enhanced levels of the CCR7 ligands CCL19 and CCL21 in HIV infection: correlation with viral load, disease progression and response to highly active antiretroviral therapy.. Aids.

[31] Damas JK, Landro L, Fevang B, Heggelund L, Tjonnfjord GE (2009). Homeostatic chemokines CCL19 and CCL21 promote inflammation in human immunodeficiency virus-infected patients with ongoing viral replication.. Clin Exp Immunol.

[32] Brenchley JM, Price DA, Schacker TW, Asher TE, Silvestri G (2006). Microbial translocation is a cause of systemic immune activation in chronic HIV infection.. Nat Med.

[33] Kadowaki N, Ho S, Antonenko S, Malefyt RW, Kastelein RA (2001). Subsets of human dendritic cell precursors express different toll-like receptors and respond to different microbial antigens.. J Exp Med.

[34] De Smedt T, Pajak B, Muraille E, Lespagnard L, Heinen E (1996). Regulation of dendritic cell numbers and maturation by lipopolysaccharide in vivo.. J Exp Med.

[35] Roake JA, Rao AS, Morris PJ, Larsen CP, Hankins DF (1995). Dendritic cell loss from nonlymphoid tissues after systemic administration of lipopolysaccharide, tumor necrosis factor, and interleukin 1.. J Exp Med.

[36] Geissmann F, Manz MG, Jung S, Sieweke MH, Merad M (2010). Development of monocytes, macrophages, and dendritic cells.. Science.

[37] Rimaniol AC, Zylberberg H, Zavala F, Viard JP (1996). Inflammatory cytokines and inhibitors in HIV infection: correlation between interleukin-1 receptor antagonist and weight loss.. Aids.

[38] Brown KN, Wijewardana V, Liu X, Barratt-Boyes SM (2009). Rapid influx and death of plasmacytoid dendritic cells in lymph nodes mediate depletion in acute simian immunodeficiency virus infection.. PLoS Pathog.

[39] Meyers JH, Justement JS, Hallahan CW, Blair ET, Sun YA (2007). Impact of HIV on cell survival and antiviral activity of plasmacytoid dendritic cells.. PLoS One.

[40] McIlroy D, Autran B, Clauvel JP, Oksenhendler E, Debre P (1998). Low CD83, but normal MHC class II and costimulatory molecule expression, on spleen dendritic cells from HIV+ patients.. AIDS Res Hum Retroviruses.

[41] Soderlund J, Nilsson C, Lore K, Castanos-Velez E, Ekman M (2004). Dichotomy between CD1a+ and CD83+ dendritic cells in lymph nodes during SIV infection of macaques.. J Med Primatol.

[42] Zimmer MI, Larregina AT, Castillo CM, Capuano S, Falo LD (2002). Disrupted homeostasis of Langerhans cells and interdigitating dendritic cells in monkeys with AIDS.. Blood.

[43] Rutella S, Danese S, Leone G (2006). Tolerogenic dendritic cells: cytokine modulation comes of age.. Blood.

[44] Qin S, Sui Y, Soloff AC, Junecko BA, Kirschner DE (2008). Chemokine and cytokine mediated loss of regulatory T cells in lymph nodes during pathogenic simian immunodeficiency virus infection.. J Immunol.

[45] Estes JD, Li Q, Reynolds MR, Wietgrefe S, Duan L (2006). Premature induction of an immunosuppressive regulatory T cell response during acute simian immunodeficiency virus infection.. J Infect Dis.

[46] Pereira LE, Villinger F, Onlamoon N, Bryan P, Cardona A (2007). Simian immunodeficiency virus (SIV) infection influences the level and function of regulatory T cells in SIV-infected rhesus macaques but not SIV-infected sooty mangabeys.. J Virol.

[47] Duttagupta PA, Boesteanu AC, Katsikis PD (2009). Costimulation signals for memory CD8+ T cells during viral infections.. Crit Rev Immunol.

[48] Beq S, Nugeyre MT, Ho Tsong Fang R, Gautier D, Legrand R (2006). IL-7 induces immunological improvement in SIV-infected rhesus macaques under antiviral therapy.. J Immunol.

[49] Karlsson I, Malleret B, Brochard P, Delache B, Calvo J (2007). Dynamics of T-cell responses and memory T cells during primary simian immunodeficiency virus infection in cynomolgus macaques.. J Virol.

[50] Herbeuval JP, Nilsson J, Boasso A, Hardy AW, Vaccari M (2009). HAART reduces death ligand but not death receptors in lymphoid tissue of HIV-infected patients and simian immunodeficiency virus-infected macaques.. Aids.

[51] Behbahani H, Landay A, Patterson BK, Jones P, Pottage J (2000). Normalization of immune activation in lymphoid tissue following highly active antiretroviral therapy.. J Acquir Immune Defic Syndr.

[52] Chehimi J, Azzoni L, Farabaugh M, Creer SA, Tomescu C (2007). Baseline viral load and immune activation determine the extent of reconstitution of innate immune effectors in HIV-1-infected subjects undergoing antiretroviral treatment.. J Immunol.

[53] Hazuda DJ, Young SD, Guare JP, Anthony NJ, Gomez RP (2004). Integrase inhibitors and cellular immunity suppress retroviral replication in rhesus macaques.. Science.

[54] Marsland BJ, Battig P, Bauer M, Ruedl C, Lassing U (2005). CCL19 and CCL21 induce a potent proinflammatory differentiation program in licensed dendritic cells.. Immunity.

[55] Hilkens CM, Kalinski P, de Boer M, Kapsenberg ML (1997). Human dendritic cells require exogenous interleukin-12-inducing factors to direct the development of naive T-helper cells toward the Th1 phenotype.. Blood.

[56] Abel K, La Franco-Scheuch L, Rourke T, Ma ZM, De Silva V (2004). Gamma interferon-mediated inflammation is associated with lack of protection from intravaginal simian immunodeficiency virus SIVmac239 challenge in simian-human immunodeficiency virus 89.6-immunized rhesus macaques.. J Virol.

[57] Li Q, Duan L, Estes JD, Ma ZM, Rourke T (2005). Peak SIV replication in resting memory CD4+ T cells depletes gut lamina propria CD4+ T cells.. Nature.

[58] Mattapallil JJ, Douek DC, Hill B, Nishimura Y, Martin M (2005). Massive infection and loss of memory CD4+ T cells in multiple tissues during acute SIV infection.. Nature.

[59] Veazey RS, DeMaria M, Chalifoux LV, Shvetz DE, Pauley DR (1998). Gastrointestinal tract as a major site of CD4+ T cell depletion and viral replication in SIV infection.. Science.

[60] Gill MA, Palucka AK, Barton T, Ghaffar F, Jafri H (2005). Mobilization of plasmacytoid and myeloid dendritic cells to mucosal sites in children with respiratory syncytial virus and other viral respiratory infections.. J Infect Dis.

[61] Li Q, Estes JD, Schlievert PM, Duan L, Brosnahan AJ (2009). Glycerol monolaurate prevents mucosal SIV transmission.. Nature.

[62] Sodora DL, Silvestri G (2008). Immune activation and AIDS pathogenesis.. Aids.

[63] Hasegawa A, Liu H, Ling B, Borda JT, Alvarez X (2009). The level of monocyte turnover predicts disease progression in the macaque model of AIDS.. Blood.

[64] Kuroda MJ (2010). Macrophages: do they impact AIDS progression more than CD4 T cells?. J Leukoc Biol.

[65] Burdo TH, Soulas C, Orzechowski K, Button J, Krishnan A (2010). Increased monocyte turnover from bone marrow correlates with severity of SIV encephalitis and CD163 levels in plasma.. PLoS Pathog.

[66] Barratt-Boyes SM, Soloff AC, Gao W, Nwanegbo E, Liu X (2006). Broad cellular immunity with robust memory responses to simian immunodeficiency virus following serial vaccination with adenovirus 5- and 35-based vectors.. J Gen Virol.

[67] Barratt-Boyes SM, Zimmer MI, Harshyne LA, Meyer EM, Watkins SC (2000). Maturation and trafficking of monocyte-derived dendritic cells in monkeys: implications for dendritic cell-based vaccines.. J Immunol.

[68] Fallert BA, Poveda S, Schaefer TM, Pfeifer ME, Sanghavi SK (2008). Virologic and immunologic events in hilar lymph nodes during simian immunodeficiency virus infection: development of polarized inflammation.. J Acquir Immune Defic Syndr.

[69] Schmittgen TD, Livak KJ (2008). Analyzing real-time PCR data by the comparative C(T) method.. Nat Protoc.

